# Jumpy and Jerky: When Peripheral Vision Faces Reverse-Phi

**DOI:** 10.1177/2041669520939107

**Published:** 2020-09-20

**Authors:** Jean Lorenceau, Patrick Cavanagh

**Affiliations:** Integrative Neuroscience and Cognition Center, CNRS UMR 8022, Université de Paris; Department of Psychology, Glendon College, Centre for Vision Research and Vision: Science to Applications York University, Toronto, ON, Canada; Department of Psychological and Brain Sciences, Dartmouth College, Hanover, NH, USA

**Keywords:** motion, reverse-phi, peripheral vision

## Abstract

When an annulus in fast apparent motion reverses its contrast over time, the foveal and peripheral percepts are strikingly different. In central vision, the annulus appears to follow the same path as an annulus without flicker, whereas in the periphery, the stimulus seems to randomly jump across the screen. The illusion strength depends on motion speed and reversal rate. Our observations suggest that it results from a balance between conflicting phi and reverse-phi motion, positional uncertainty, and attention. In addition to illustrating the differences between central and peripheral motion processing, this illusion shows that both discrete positional sampling and motion energy combine to generate motion percepts, although with eccentricity dependent weights that are themselves affected by attention.

Stuart Anstis (1970) discovered that when a stimulus displaces and reverses contrast at the same time, the apparent motion is in a direction opposite to the displacement. Movie 1 shows an example of this effect: the global perceived rotations of the rings are in a direction opposite to the veridical direction, as it can be seen when tracking a single element, although sustained fixation can entail a variety of percepts. The perception of this *reverse-phi* motion depends on motion speed and reversal rate (Anstis & Rogers, 1986; Sato, 1989). Single neurons in macaque V1 (Livingstone & Conway, 2003) and MT (Krekelberg & Albright, 2005) also invert their preferred direction when stimulated by moving stimuli with alternating contrast polarity, indicating that “direction-selective cells are generated by combining spatially and temporally offset inputs that are linear with respect to contrast, changing their firing rate in opposite directions for stimuli of opposite contrasts” (Livingstone & Conway, 2002, 2003; also see Mo & Koch, 2003).

**Movie 1.**
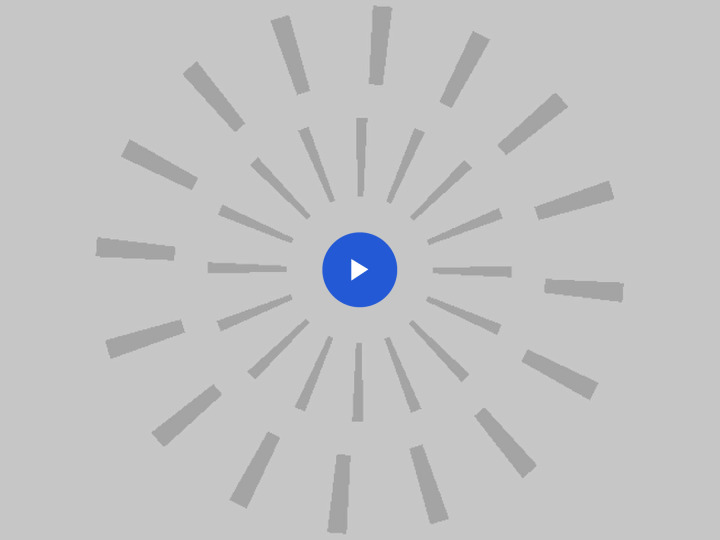
Demonstration of Reverse-Phi Motion. The global impression of motion is a clockwise rotation in the inner ring and counterclockwise in the outer ring, although each bar of the patterns moves counterclockwise and clockwise, respectively, as can be seen when attending to a single element. The reverse motion is only seen if there are enough pattern elements so that the reversal can have a plausible target. If there is only one bar flickering and moving, it is seen to move in its veridical direction. Also note that sustained fixation entrains perceptual instabilities: the motion may stop or reverse periodically.

Here, we report that when seen in far periphery (>10°–50°), a contrast reversing annulus moving quickly along a circular path generates a jerky percept, with large and sudden jumps in position, with sometimes smooth spiral motion of small amplitude, whose speed differs from the veridical path and speed (hereafter the *Jumpy-Jerky illusion*, Movie 2). These random jumps and local motions can even make it difficult to determine whether the motion is clockwise or anticlockwise. In central vision, the motion path is easily seen and compares well with an annulus moving without contrast reversals. This illusion is best seen for contrast reversals rates between 6 and 20 Hz. Although it is more compelling at higher rates in this range, it vanishes above 30 Hz. The effect does not strongly depend on contrast, but is less salient or even disappears if the contrast alternations are unbalanced, or always positive or negative. (Thus, the Movies’ appearance may depend on the display’s gamma correction). Adding four dots surrounding the annulus and moving along a circular trajectory identical to the annulus motion reduces, but does not abolish, the effect.

**Movie 2.**
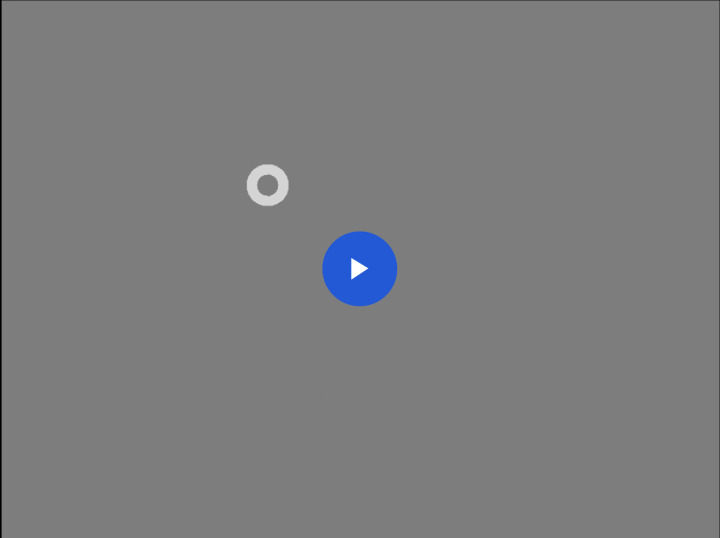
Example of the Jumpy-Jerky motion. Directing the gaze to the moving stimulus or to eccentric fixation dramatically changes the motion percept. The motion path appears highly jerky when the gaze is off screen (up to a limit where the stimulus is hardly seen). In addition, the perceived motion is highly sensitive to smooth eye movement, for instance when tracking one’s finger moving up and down, suggesting that the visual system cannot correct for eye movements with a combination of phi and reverse-phi motion, contrary to what occurs with phi-motion only. The perceived motion further changes with different eccentricities, and its Jumpy-Jerky appearance is stronger at very large eccentricities. As the stimuli were displayed on a large 75 Hz screen, it might be necessary to download and watch the movies at full scale to maximize the illusion.

These observations suggest that the Jumpy-Jerky illusion results from an eccentricity-dependent conflict between phi and reverse-phi motion signaling opposite motion directions. Apparently, in central vision, strong position signals overcome the reverse-phi signals and the veridical motion is seen. In the periphery, the positional uncertainty is larger (perhaps due to larger receptive fields) and the reverse motion signals now contribute more effectively to estimates of the annulus’s position. As a result, it jumps away from its true path until a position error exceeds the positional uncertainty and it then jumps back toward its true location. It is worth noting that the Jumpy-Jerky motion appearance depends on which part of the periphery is stimulated, as can be tested by fixating different eccentric locations, in monocular or binocular vision. This suggests that motion processing in the periphery is itself heterogeneous with regard to phi and reverse-phi motion.

During our explorations, we observed that the strength of the illusion diminishes when attending to the stimulus while keeping an eccentric fixation (covert tracking). In this case, its perceived path resembles that of a stimulus lacking contrast reversals. This suggests that attention has an asymmetrical effect on phi and reverse-phi and selectively decreases the contribution of reverse-phi signals (Treue & Maunsell, 1996). To demonstrate this, we introduce a second flickering annulus with a different motion trajectory (Movie 3). Now, the motion of the attended annulus seems veridical while the untracked stimulus jumps around randomly. Switching attention from one annulus to the other also switches the percepts. At very high speeds, covert tracking becomes difficult such that both stimuli appear jerky and jumpy. Movie 4 provides examples of different contrast reversal rates, motion speeds, and the presence of a moving reference frame to illustrate their respective effects.

**Movie 3.**
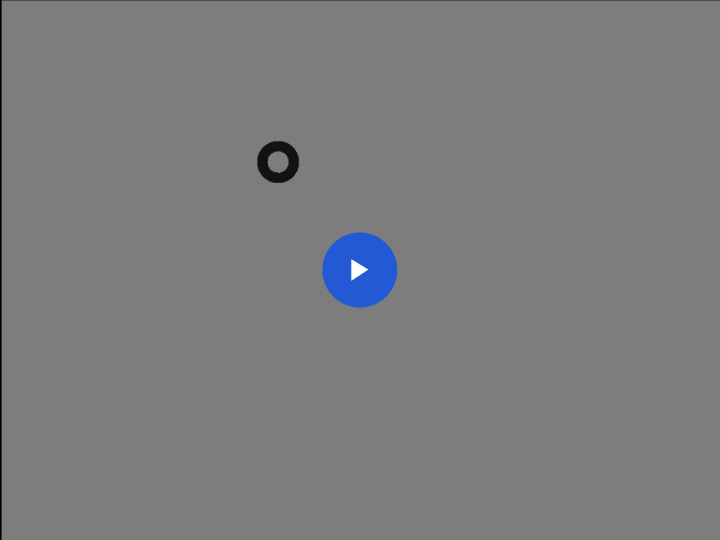
Double Jumpy-Jerky Motion and Attention. In Movie 2, it is possible to covertly track the stimulus, which then appears to move more or less smoothly along the veridical circular path. In this example, with two annuli moving along opposite paths, covertly attending to one stimulus during eccentric fixation reduces the Jumpy-Jerky illusion for the attended, but not the unattended stimulus, that appears to make jerky jumps all around.

**Movie 4.**
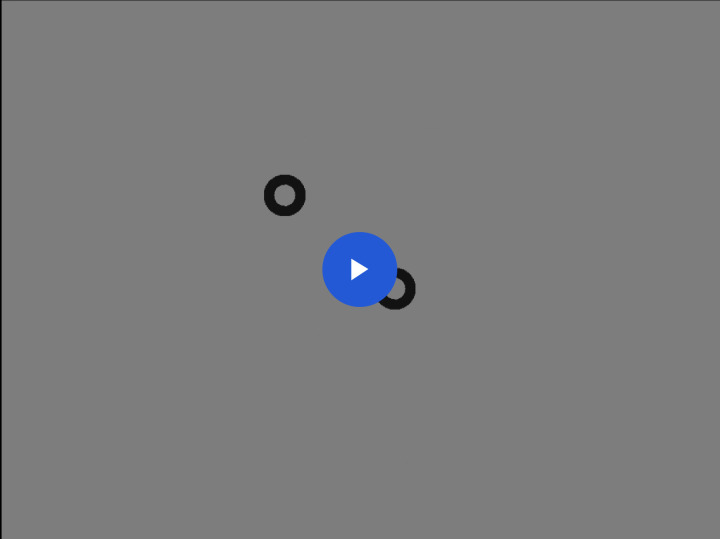
Combines Several Motion Stimuli With Different Parameters: Rate of Contrast Reversals, Speed, and Presence of a Reference Frame. The movie starts with the Jumpy- Jerky motion used in Movie 3; the contrast reversals of the bottom annulus then switches to a slow rate, before the two annuli have a slow reversal rate; the motion then shifts to a high speed and high reversal rates. The last part shows the effect of adding four white dots around the annuli, moving as in Movie 3, to illustrate the influence of a reference frame.

If phi and reverse-phi perfectly canceled over time, the stimulus should appear stationary until a position error drives a correction to the veridical location, resulting in a sudden jump, which should occur periodically. However, the Jumpy-Jerky percept suggests that the conflict between phi and reverse-phi is resolved in an erratic way. This could be due to positional and speed uncertainty changing with eccentricity (Hassan et al., 2016), to unbalanced motion energy and timing of phi and reverse-phi *events* (on each frame for phi motion, and with longer, rate dependent, intervals for reverse-phi), or to the wide distribution of motion directions present during the circular motion.

This compelling phenomenon appears in peripheral but not in central vision, so it cannot results from eye movements that induce similar retinal slips for central and peripheral stimuli. An (in)attentional sampling of this motion stimulus, possibly related to cortical rhythms (VanRullen & Koch, 2003), is also unlikely to account for this illusion, as this should also hold in central vision, which is not the case.

The phenomenal description presented here could be extended to experimentally assess the effects of stimulus shape, size and contrast, motion anisotropies in the visual field, attention and eye movements, as well as to document idiosyncrasies that may exist, in order to shed light on the underlying mechanisms.
